# Leaf Nitrogen Allocation Trade-Offs Promote Efficient Utilization of Different Nitrogen Forms in *Hemarthria altissima*

**DOI:** 10.3390/biology14091260

**Published:** 2025-09-12

**Authors:** Nan Xu, Xiaowei Wei, Ju Zhang, Mingyue Sun, Jinwei Zhang, Zihao Zhao, Xuechen Yang

**Affiliations:** 1 Key Laboratory of Heilongjiang Province for Cold-Regions Wetlands Ecology and Environment Research, Harbin University, Harbin 150086, China; xunan0451@126.com; 2 Jilin Provincial Key Laboratory for Plant Resources Science and Green Production, Jilin Normal University, Siping 136000, China; weixiaowei@jlnu.edu.cn (X.W.); 13894155011@163.com (J.Z.); 18543444519@163.com (M.S.); 13397005645@163.com (Z.Z.); 3 Department of Grassland Science, College of Animal Science and Technology, Northeast Agricultural University, Harbin 150086, China; zhangjw133@nenu.edu.cn; 4 State Key Laboratory of Ecological Safety and Sustainable Development in Arid Lands, Xinjiang Institute of Ecology and Geography, Chinese Academy of Sciences, Urumqi 830011, China

**Keywords:** ammonium nitrogen, nitrate nitrogen, photosynthetic nitrogen-use efficiency, leaf nitrogen allocation, *Hemarthria altissima*

## Abstract

The sharp increase in atmospheric nitrogen deposition has had profound effects on nitrogen availability and the photosynthetic capacity of terrestrial plants. Consequently, understanding the intricate trade-off between nitrogen sources and their allocation within leaves is essential for unraveling the photosynthetic responses of grassland ecosystems to nitrogen deposition. In a series of field experiments, the effects of different nitrogen forms (ammonium and nitrate nitrogen) on nitrogen assimilation and allocation in the C_4_ plant *Hemarthria altissima* were thoroughly investigated. Towards the end of the growing season, *H. altissima* was observed to exhibit high photosynthetic efficiency. Ammonium nitrogen treatment notably enhanced photosynthetic nitrogen use efficiency (PNUE) by modifying the nitrogen allocation within the leaf’s photosynthetic apparatus and leaf area, leading to a significant improvement in photosynthetic efficiency and biomass accumulation. Under ammonium nitrogen treatment, *H. altissima* directed more nitrogen toward its carboxylation process and other protein-related functions to increase carboxylation efficiency, thereby facilitating the accumulation of photosynthetic products. In contrast, under nitrate nitrogen treatment, the plant balanced growth and light absorption by allocating nitrogen to leaf light-capturing proteins. The application of both ammonium and nitrate nitrogen resulted in increased nitrogen content in the soil, as ammonium nitrogen is converted to nitrate nitrogen through nitrification. The net photosynthetic rate (*A_n_*), nitrogen allocation to photosynthetic components (*N_psn_*), and chlorophyll content per unit area (*Chl_area_*) were all significantly and positively correlated with photosynthetic nitrogen use efficiency (PNUE). Notably, under the sole NH_4_^+^ treatment, nitrogen allocation to the photosynthetic components increased, which enhanced the NPQ and ETR in *H. altissima* leaves. These findings suggest that *H. altissima* preferentially utilizes ammonium nitrogen from the soil, optimizing its PNUE and biomass accumulation through a strategic allocation of nitrogen within its leaves. Further investigation is needed to explore how these nitrogen allocation strategies may vary under different environmental conditions and how they influence ecosystem-level productivity.

## Simple Summary

Global environmental change has intensified nitrogen deposition, resulting in alterations to the availability and balance of nitrate (NO_3_^−^), ammonium (NH_4_^+^), and their mixtures in the soil. These nitrogen forms are known to exert distinct effects on plant physiology, photosynthesis, and nutrient-use strategies. While nitrate is often associated with enhanced plant growth, ammonium may induce stress or toxicity, depending on the species and environmental conditions. A comprehensive understanding of plant responses to different nitrogen forms is considered essential for maintaining grassland productivity and ecological resilience. In this study, the effects of nitrate, ammonium, and their combination on *Hemarthria altissima*, a valuable forage species in grassland ecosystems, were investigated. The influence of different nitrogen forms on soil nitrogen availability, photosynthetic performance, nitrogen allocation within the photosynthetic system, and photosynthetic nitrogen-use efficiency (PNUE) were analyzed. Key adaptive mechanisms of *H. altissima* under varying nitrogen conditions were revealed, contributing to strategies for sustainable grassland management under the pressures of global environmental change.

## 1. Introduction

Nitrogen (N) plays a pivotal role in supporting plant growth and development, with a profound influence on ecosystem productivity. For plants, nitrogen is absorbed predominantly in the forms of ammonium (NH_4_^+^) and nitrate (NO_3_^−^), which are not only vital for metabolic processes but also represent the primary forms of nitrogen loading resulting from atmospheric deposition [1]. Grasses, especially forage species, exhibit a high degree of plasticity in their ability to absorb and utilize these nitrogen forms. This ability allows them to thrive in a wide range of environmental conditions, adjusting their nitrogen acquisition strategies based on the relative availability of NH_4_^+^ and NO_3_^−^ in the soil [2]. Such flexibility is crucial for maintaining high biomass production in grasslands, which is essential for both agricultural productivity and ecological balance.

The availability of ammonium nitrogen (NH_4_^+^) and nitrate nitrogen (NO_3_^−^) in the soil directly impacts several physiological processes, from nitrogen uptake to photosynthesis and overall plant biomass. Grasses often show a stronger growth response to NH_4_^+^, as it is energetically less expensive to assimilate compared to NO_3_^−^. This is particularly advantageous under conditions where nitrogen availability is low or sporadic. However, while NH_4_^+^ provides a readily available nitrogen source, its accumulation can have toxic effects, particularly at higher concentrations. Excess NH_4_^+^ can lead to disruptions in ionic homeostasis, resulting in oxidative stress and metabolic disturbances, which impair plant growth and development [3]. Such imbalances can affect cellular respiration, nutrient uptake, and even chlorophyll synthesis, resulting in stunted growth and reduced photosynthetic efficiency.

On the other hand, nitrate (NO_3_^−^), while requiring more energy to assimilate, offers a more stable nitrogen source for grasses when available in adequate amounts. NO_3_^−^ uptake by grasses is generally regulated through high-affinity and low-affinity transporter systems in roots, which allow plants to efficiently exploit soil nitrogen at varying concentrations [4]. The presence of NO_3_^−^ in the soil also enhances the assimilation of NH_4_^+^, particularly in the roots, as nitrate reductase activity increases in response to nitrate uptake, facilitating the processing of both nitrogen forms [5]. This cross-regulation between NH_4_^+^ and NO_3_^−^ highlights the complex relationship between nitrogen forms and their collective impact on plant growth. Notably, grasses that have access to both nitrogen forms tend to exhibit improved nitrogen-use efficiency (NUE), enhancing their ability to allocate nitrogen toward biomass production and photosynthetic capacity [6].

The relationship between carbon and nitrogen metabolism is intricately connected at multiple scales, from leaf to whole-plant levels. In grasses, nitrogen influences both carbon assimilation and photosynthetic efficiency, which are critical for overall growth and productivity. Nitrogen plays a central role in the synthesis of enzymes involved in carbon fixation and electron transport. Specifically, nitrogen is a key component of Rubisco (ribulose-1,5-bisphosphate carboxylase/oxygenase), the enzyme responsible for carbon fixation in the C_4_ photosynthetic pathway of grasses, which is highly efficient in tropical and subtropical regions [7]. Thus, nitrogen availability directly influences photosynthetic nitrogen-use efficiency (PNUE), a critical trait for the productivity of forage grasses. Even small fluctuations in nitrogen availability can have a profound impact on carboxylation efficiency and light-harvesting efficiency, with significant consequences for plant biomass accumulation [8].

In the absence of sufficient nitrogen, grasses experience impaired photosynthesis, leading to reduced growth and altered nitrogen allocation across plant tissues. For instance, during nitrogen deficiency, grasses may reallocate nitrogen away from photosynthetic proteins and into structural components, including cell walls and defensive molecules, as a stress response to optimize growth under limited nitrogen availability [9]. This shift in nitrogen allocation impacts leaf mass per area (LMA) and the overall leaf economics spectrum, with native species typically investing more nitrogen into defensive proteins than invasive species, which prioritize photosynthetic machinery [10].

Gramineous forage grasses, such as maize, sorghum, and ryegrass, adapt their nitrogen-use strategies to optimize growth under variable soil nitrogen conditions. For example, under low-nitrogen conditions, some grasses shift nitrogen allocation toward maintaining critical nitrogen enzymes and bioenergetic pathways, ensuring they can sustain electron transport and cellular respiration [11]. In contrast, excessive nitrogen supply, particularly from ammonium, can lead to a shift toward greater root growth and higher allocation to structural components such as cell walls, rather than photosynthetic tissues, which ultimately reduces the efficiency of carbon fixation and slows growth [12].

Understanding the interaction between soil nitrogen forms and grass growth is essential for agricultural management, particularly in the context of optimizing fertilizer applications for forage production. By balancing ammonium and nitrate inputs, farmers and land managers can enhance forage yield, improve nutrient-use efficiency, and minimize nitrogen leaching and environmental pollution. Furthermore, identifying species that are more resilient to fluctuations in nitrogen availability could lead to more sustainable farming practices, especially in regions where nitrogen inputs are becoming increasingly unpredictable due to environmental changes and agricultural practices. The following hypothesis is proposed: (1) Increasing nitrogen deposition (inorganic nitrogen forms) will lead to enhanced nitrogen assimilation and improved nitrogen-use efficiency in *H. altissima*, particularly in environments with high ammonium nitrogen availability. (2) The adaptive response of *H. altissima* to rising nitrogen deposition will be characterized by increased nitrogen allocation to photosynthetic components, improving photosynthetic efficiency and biomass production, but potentially leading to trade-offs in other physiological processes like growth and root development.

## 2. Materials and Methods

### 2.1. Study Area and Growth Conditions

The field experiment was conducted at the Jilin Songnen Grassland Ecosystem National Observation and Research Station (44°34′ N, 123°31′ E), China, within a temperate continental monsoon climate zone. The region has hot, rainy summers and cold, dry winters. Soil in the 0–20 cm layer exhibited a pH of 8.68, EC of 78.76 μS·cm^−1^, total N of 1.02 g·kg^−1^, total P of 0.66 g·kg^−1^, organic C of 6.37 g·kg^−1^, NH_4_^+^-N of 1.23 mg·kg^−1^, and NO_3_^−^-N of 1.89 mg·kg^−1^. Annual mean temperature ranged from 4.6 to 6.5 °C, with total precipitation between 280 and 620 mm, mostly falling between June and September [13]. The pot experiment used wind-sand soil (3.5 kg pot^−1^) and followed a completely randomized block design with six replicates.

*Hemarthria altissima* (Poir.) Stapf & C. E. Hubb. is a perennial C_4_ forage grass in the Poaceae family, known for its long, horizontally creeping rhizomes and strong nutritional and reproductive capacities. It is widely distributed across the natural meadows of the Songnen Plain, where it often dominates or serves as a key companion species in plant communities [14]. On May 10th, shoots of *H. altissima* were transplanted into plastic pots, collected from the eastern Eurasian meadow steppe. Based on the population density during the green period (May 10th–May 30th), six individuals per pot were planted in monoculture, and plots were harvested on September 15th. Nitrogen treatments included: no nitrogen (N0), sole NH_4_^+^-N [as (NH_4_)_2_SO_4_ (Sinopharm Chemical Reagent Co., Ltd., Shanghai, China)] (AN), sole NO_3_^−^-N [as Ca(NO_3_)_2_ (Sinopharm Chemical Reagent Co., Ltd., Shanghai, China)] (NN), and a 1:1 mixture of NH_4_^+^-N and NO_3_^−^-N (NAN), with 10 g N m^−2^ applied in two equal doses on May 30th and June 15th. Dicyandiamide (DCD, 98.0%) (Sinopharm Chemical Reagent Co., Ltd., Shanghai, China) was added to the AN and NAN treatments to inhibit nitrification, at 10 mg m^−2^ y^−1^ and 5 mg m^−2^ y^−1^, respectively. Apply Hogland nutrient solution (KH_2_PO_4_ 2.5 mmol L^−1^, MgSO_4_·7H_2_O 2 mmol·L^−1^, H_3_BO_3_ 40 μmol·L^−1^, MnCl_2_·4H_2_O 10 μmol·L^−1^, ZnSO_4_·7H_2_O 0.8 μmol·L^−1^, CuSO_4_·5H_2_O 0.4 μmol·L^−1^, Na_2_MoO_4_·2H_2_O 0.2 μmol·L^−1^, EDTA-Fe 20 μmol·L^−1^) (Tianjin Huasheng Tianhe Chemical Trading Co., Ltd., Tianjin, China) once a month, 200 mL pot^−1^ each time, all treatments received additional fertilizers (P, K, S) and micronutrients (Zn, B, Mn, Mo, Cu, Fe) to avoid nutrient limitations. Weeds, insects, and diseases were controlled, and the plots were exposed to natural precipitation with minimal irrigation. Harvesting occurred at the post-fruiting stage on September 15th.

### 2.2. Gas Exchange Measurements and Chlorophyll Fluorescence

From August 2nd to 10th, leaf assimilation rate (*A_n_*, μmol m^−2^ s^−1^), stomatal conductance (*G_s_*, mmol m^−2^ s^−1^), internal CO_2_ (*C_i_*, μmol mol^−1^), and water use efficiency (*WUE*, %) were measured using a CIRAS-3 portable photosynthesis system (PP Systems, Amesbury, MA, USA) at 25 °C, with a CO_2_ concentration of 400 μmol mol^−1^ and a 500 μmol s^−1^ flow rate. The photosynthetic photon flux density (PPFD) was set to 2000 μmol m^−2^ s^−1^ [15]. For the rapid A/C_i_ response curve [16], CO_2_ partial pressure was varied from 50 to 1600 μmol mol^−1^. Gas exchange measurements were taken from the 2nd and 3rd leaves of the shoot, between 8:00 AM and 3:00 PM, with six replicates per pot.

The maximum rate of Rubisco carboxylation (*V_cmax_*, μmol m^−2^ s^−1^) and the maximum rate of electron transport (*J_max_*, μmol m^−2^ s^−1^) were derived from *A*_n_/*C_i_* curve data, and the values were fitted using the models of [17,18]. The calculations followed these formulas:

(1) $$V_{cmax}=\frac{\left(R_{d}+A_{n}\right)[C_{i}+K_{C}(1+\frac{O}{K_{0}})]}{\left(C_{i}-\Gamma^{*}\right)}$$

(2) $$J_{max}=\frac{4(R_{d}+A_{n})(C_{i}+2\Gamma^{*})}{\left(C_{i}-\Gamma^{*}\right)}$$

where *R_d_* represents the mitochondrial respiration rate in the light (μmol m^−2^ s^−1^), *K_c_* and *K_o_* are the Michaelis constants for carboxylation and oxygenation, respectively, *O* is the intercellular oxygen concentration (approximately 210 mmol mol^−1^), and Γ* is the CO_2_ compensation point in the absence of respiration (μmol mol^−1^). Additionally, the values for *Kc*, *Ko* and Γ* were determined using the temperature-dependent functions outlined [18].

The following day, chlorophyll fluorescence measurements were taken using the IMAGING PAM M-series (Walz, Effeltrich, Germany). Prior to the measurements, samples were dark-adapted for 30 min. The maximum quantum yield of PSII (F_v_/F_m_), the effective quantum yield of PSII (φPSII), the non-photochemical quenching coefficient (NPQ), and the electron transport rate (ETR, μmol e^−1^ s^−1^ m^−2^) were referred to previous analytical methods [15]. After measuring the chlorophyll fluorescence parameters, leaf area was determined using a portable leaf area meter (AM350, ADC Bio Scientific Ltd., Herts, UK).

### 2.3. Sample Collection and Chemical Analyses

On September 15, soil samples were collected using the five-point method. After passing through a 100-micron sieve and removing the roots, the moist soil was separated into two parts using a 1-mm sieve. One part was kept fresh (4 °C) for the analysis of ammonium nitrogen and nitrate nitrogen in the soil. The other half was air-dried naturally for the determination of total nitrogen (TN) in the soil. The total nitrogen content was determined using an elemental analyser (vario EL cube, Elementar, Langenselbold, Germany). A 10 g fresh soil sample was placed in a 100-mL flask containing 50 mL of 2 M KCL to measure extractable ammonium and nitrate. The sample was shaken at 180 rpm for 1 h on an orbital shaker. Afterward, the extract was filtered through a 0.45 μm nylon net Millipore filter (prewashed with 2 M KCL). The filtered extracts were stored in plastic vials and frozen at −20 °C for no longer than one week before analysis. Soil ammonium and nitrate concentrations were measured using an Alliance Flow Analyser (Alliance Flow Analyser, Futura, Frépillon, France). Dissolved inorganic nitrogen (DIN) was calculated as the sum of the extractable ammonium and nitrate in the soil.

Two leaves from each plant were collected, immediately frozen in liquid nitrogen, and stored at −80 °C for biochemical analysis. Additionally, two leaves were subjected to enzyme activity inhibition by heating at 105 °C for 30 min, then dried to a constant weight at 65 °C. These leaves were used for biomass measurement and total nitrogen content analysis (*N_m_*, mg g^−1^) using an Elementar Vario EL Cube (Elementar, Langenselbold, Germany). Leaf mass per unit leaf area (*LMA*, g m^−2^) and leaf nitrogen content per unit leaf area (*N_area_*, g m^−2^) were calculated using the formula: *N_area_* = *N_m_* × *LMA*. Chlorophyll content per leaf mass (*Chl_m_*, mg g^−1^) was quantified by extracting 0.1 g leaf tissue in ethanol, and absorbance was measured at 645 nm and 663 nm using a spectrophotometer (UVmini-1240, Shimadzu, Kyoto, Japan), following the method of Wellburn (1994) [19]. Chlorophyll content was calculated as follows:

(3) $${Chl}_{a}=12.56\times A_{665}-2.71\times A_{645}$$

(4) $${Chl}_{b}=22.65\times A_{645}-4.35\times A_{663}$$

(5) $${Chl}_{m}={Chl}_{a}+{Chl}_{b}$$

Chlorophyll content per leaf area (*Chl_area_*) was determined by multiplying *Chl_m_* by *LMA*.

To quantify nitrate nitrogen and ammonium nitrogen content, 2.0 g of lyophilized leaf samples were incubated with 10 mL distilled water, boiled for 1 h, and filtered to obtain the crude extract. Nitrate concentration (NO_3_^−^) was measured using the salicylic acid chromogenic method [20], while ammonium concentration (NH_4_^+^) was determined by the phenol-hypochlorite method [21]. Free amino acids were analyzed using the ninhydrin colorimetric method [22].

For the analysis of different nitrogen forms, the procedure described by Takashima et al. (2004) and Onoda et al. (2017) was followed with some modifications [7,8]. Leaves were powdered in liquid nitrogen and homogenized in 2 mL of Na-phosphate buffer (pH 7.5, 100 mmol L^−1^), followed by washing in a centrifuge tube. This procedure was repeated three times. The homogenates were centrifuged at 12,000× *g* for 10 min at 4 °C, and the supernatant was collected as soluble protein. The pellet was washed with 1 mL of phosphate buffer containing 3% sodium dodecyl sulfate (SDS), and after heating at 90 °C for 5 min, it was centrifuged again. This step was repeated six times, collecting the SDS-soluble protein. The residue was regarded as cell wall protein, washed with ethanol, and filtered onto quantitative filter paper. The supernatant was precipitated with 10% trichloroacetic acid (TCA) by heating at 85 °C for 5 min. The precipitate was filtered, washed with ethanol, and dried at 85 °C before being analyzed for nitrogen content by the Elementar Vario EL Cube.

Finally, the enzyme activities of ribulose-1,5-bisphosphate carboxylase/oxygenase (Rubisco), phosphoenolpyruvate carboxylase (PEPC), nitrate reductase (NR), nitrite reductase (NiR), glutamine synthetase (GSⅠ), and Glutamate synthase (GOGAT)were determined in frozen leaves using specific ELISA kits (Shanghai Enzyme Biotechnology Co., Ltd., Shanghai, China), following the manufacturer’s instructions.

### 2.4. Estimation of Nitrogen Allocation in the Photosynthetic Machinery and Its Efficiency in Photosynthetic Nitrogen Utilization

According to the LUNA model [23,24], leaf photosynthetic nitrogen is divided into three main components: the fractions allocated to the carboxylation system (*PN_C_*, g g^−1^), electron transport components (*PN_B_*, g g^−1^), and light-harvesting components (*PN_L_*, g g^−1^). These components were calculated as follows:

(6) $${PN}_{C}=\frac{V_{cmax}}{6.25\times V_{cr}\times N_{area}}$$

(7) $${PN}_{B}=\frac{J_{max}}{8.06\times J_{mc}\times N_{area}}$$

(8) $${PN}_{C}=\frac{C_{c}}{C_{B}\times N_{area}}$$

where 6.25 (g Rubisco g^−1^ N) is the conversion factor for Rubisco N at 25 °C [25], and *V_cr_* = 20.78  (μmol CO_2_ g^−1^ Rubisco s^−1^) [23]. The factor 8.06 is the N conversion coefficient for cytochrome [26], and *J_mc_* = 155.65  (μmol e^−1^ μmol cytochrome f s^−1^) at 25 °C [23,24]. *C_c_* refers to leaf chlorophyll content (mmol g^−1^), and *C_b_* is the chlorophyll binding to light-harvesting components (2.15 mmol g^−1^ N) [27].

The fractions of leaf N allocated to the thylakoid (*PN_B_* + *PN_L_*, g g^−1^) and to the photosynthetic apparatus (*PN_PSN_*, g g^−1^) are the sums of *PN_B_* and *PN_L_*, and of *PNc*, *PN_B_*, and *PN_L_*, respectively. The N contents in the carboxylation system (*Nc*, g m^−2^), bioenergetics (*N_B_*, g m^−2^), light-harvesting system (*N_L_*, g m^−2^), and the entire photosynthetic apparatus (*N_psn_*, g m^−2^) were calculated by multiplying *PN_C_*, *PN_B_*, *PN_L_*, and *PN_psn_* by *N_area_*. The remaining leaf N was considered as other N. Photosynthetic N use efficiency (PNUE, μmol g N^−2^ s^−1^) was determined as the ratio of *A_n_* to *N_area_* [28].

### 2.5. Statistical Analysis

All data were examined for a normal distribution (Kolmogorov–Smirnov test) and homogeneity of variance (Levene’ s test) and conducted using R version 4.5.1 (R Core Team, 2025). Analyses were performed using the “Fisher’s Least Significant Difference” function from “agricolae” package, differences were considered significant for *p* < 0.05. For correlation analysis, the “pearson” function in the “gpairs” and “ggpmisc” packages was utilized, and the “ggplot2” package was employed for creating graphics.

## 3. Results

### 3.1. Soil Nitrogen Characteristics

**Figure 1:**
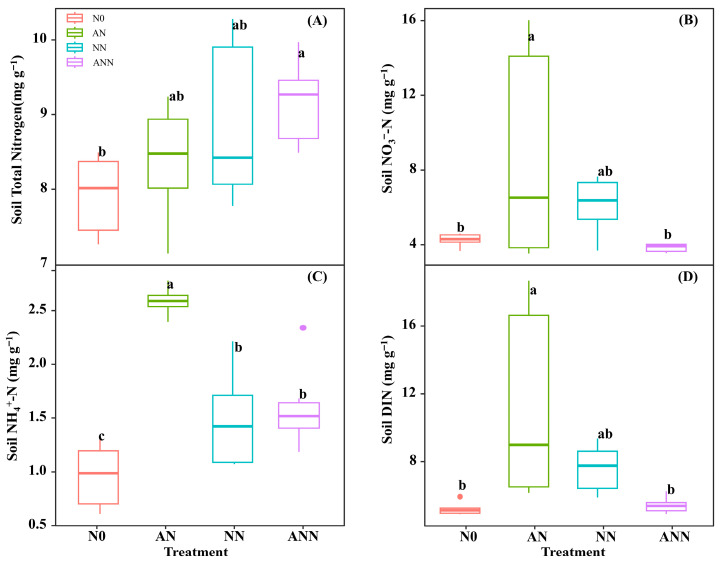
Effect of nitrogen forms treatments on soil total nitrogen (**A**), soil NO_3_^−^-N (**B**), soil NH_4_^+^-N (**C**) and soil DIN (**D**) in *H. altissima*. Different lower-case letters indicate significant differences between the measuring dates under the unfertilized (N0) treatment and the fertilized (AN, NN, ANN) treatment, respectively (*p* < 0.05) (n = 6).

The effects of N0, AN, NN, and ANN on soil total nitrogen, soil NO_3_^−^ -N, soil NH_4_^+^-N and soil DIN were significant (*p* < 0.05) (Figure 1). The soil total nitrogen of the AN, NN and ANN treatments were significantly higher than N0 treatment (*p* < 0.05) (Figure 1A). The soil NO_3_^−^ -N, soil NH_4_^+^-N and soil DIN of the AN treatment were significantly higher than those of the N0, NN and ANN treatments (*p* < 0.05) (Figure 1B–D).

### 3.2. Leaf Nitrogen Assimilation Enzyme Activity

**Figure 2:**
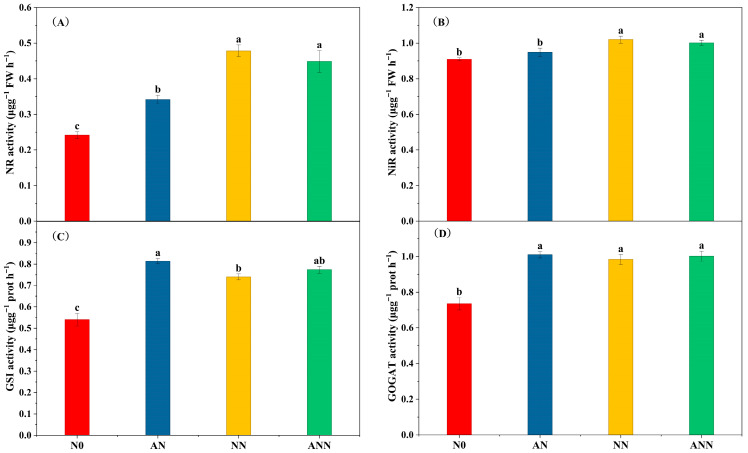
Effect of nitrogen forms treatments on NR activity (**A**), NiR activity (**B**), GSⅠ activity (**C**) and GOGAT activity (**D**) in *H. altissima*. Different lower-case letters indicate significant differences between the measuring dates under the unfertilized (N0) treatment and the fertilized (AN, NN, ANN) treatment, respectively (*p* < 0.05) (n = 6).

Nitrogen absorption from the soil depends on the activity of enzymes involved in nitrogen metabolism. NR and NiR activities were stimulated in the NN and ANN treatments; conversely, they were inhibited in the AN treatment (*p* < 0.05) (Figure 2A,B). The GSⅠ activity of the AN treatment was significantly higher than N0 and NN treatments (*p* < 0.05) (Figure 2C). The GOGAT activity of the AN, NN and ANN treatments were significantly higher than N0 treatment (*p* < 0.05), but the GOGAT activity in the AN, NN, and ANN treatments showed no significant differences (Figure 2D).

### 3.3. Leaf Gas Exchange Parameters and Morphological Characteristics

**Figure 3:**
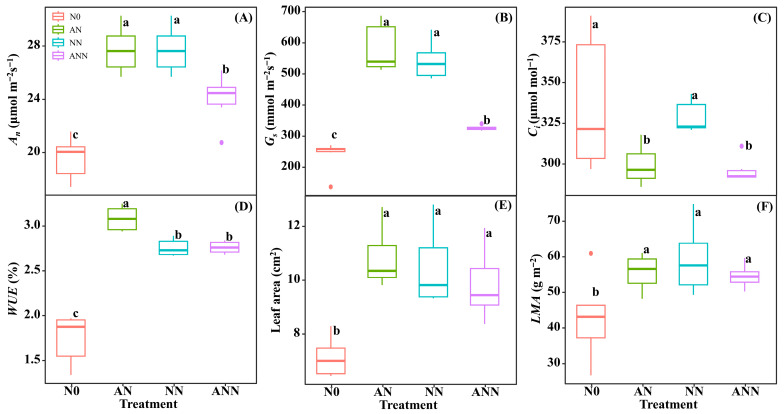
Effect of nitrogen forms treatments on net CO_2_ assimilation rate (*A_n_*) (**A**), stomatal conductance (*G_s_*) (**B**), internal CO_2_ (*C_i_*) (**C**), water use efficiency (*WUE*) (**D**), leaf area (**E**) and leaf mass area (*LMA*) (**F**) in *H. altissima*. Different lower-case letters indicate significant differences between the measuring dates under the unfertilized (N0) treatment and the fertilized (AN, NN, ANN) treatment, respectively (*p* < 0.05) (n = 6).

The *A_n_* and *G_s_* of the AN and NN treatments were significantly higher than N0 and ANN treatments, and the *A_n_* and *G_s_* of the ANN were significantly higher than N0 (*p* < 0.05) (Figure 3A,B). The *C_i_* of the AN and ANN treatments were significantly lower than N0 and NN treatments (*p* < 0.05) (Figure 3C). The *WUE* of the AN treatment was significantly higher than N0, NN and ANN treatments (*p* < 0.05) (Figure 3D). The leaf area and LMA of the AN, NN and ANN treatments were significantly higher than N0 treatment (*p* < 0.05) (Figure 3E,F).

### 3.4. Leaf Photosynthetic Pigment

The *Chl_a_* of the AN, NN and ANN treatments were no significant difference (Table 1). The *Chl_b_* of the AN treatment was 86.53% and 40.58% higher than N0 and NN treatments (*p* < 0.05). The *Chl_m_* of the AN treatment were 29.55%, 15.54% and 20.42% higher than N0, NN and ANN treatments (*p* < 0.05). The *Chl_area_* of the AN, NN and ANN treatments were significantly higher than N0 treatment (*p* < 0.05) (Table 1).

### 3.5. Leaf Photosynthetic Efficiency and Photosynthetic Nitrogen Utilization Efficiency

**Figure 4:**
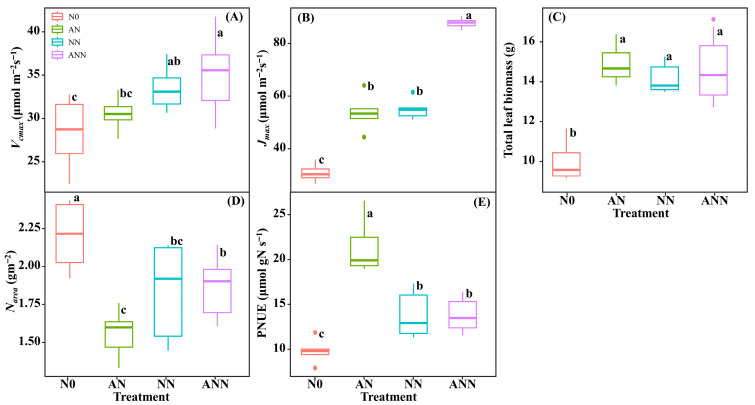
Effect of nitrogen forms treatments on maximum carboxylation rate (*V_cmax_*) (**A**), maximum photoelectron transfer rate (*J_max_*) (**B**), total leaf biomass (**C**), area-based nitrogen content (*N_area_*) (**D**) and photosynthetic N use efficiency (PNUE) (**E**) in *H. altissima*. Different lower-case letters indicate significant differences between the measuring dates under the unfertilized (N0) treatment and the fertilized (AN, NN, ANN) treatment, respectively (*p* < 0.05) (n = 6).

The *V_cmax_* and *J_max_* of the ANN treatment was significantly higher than other treatments, and the *V_cmax_* and *J_max_* of the AN and NN were significantly higher than N0 (*p* < 0.05) (Figure 4A,B). The total leaf biomass of the AN, NN and ANN treatments were significantly higher than N0 treatment (*p* < 0.05) (Figure 4C). The *N_area_* of the N0 treatment was significantly higher than AN, NN and ANN treatments (*p* < 0.05) (Figure 4D). The PNUE of the AN treatment was significantly higher than N0, NN and ANN treatments, and the PNUE of the NN and ANN were significantly higher than N0 (*p* < 0.05) (Figure 4E).

### 3.6. Leaf Photosynthetic Enzyme Activity

**Figure 5:**
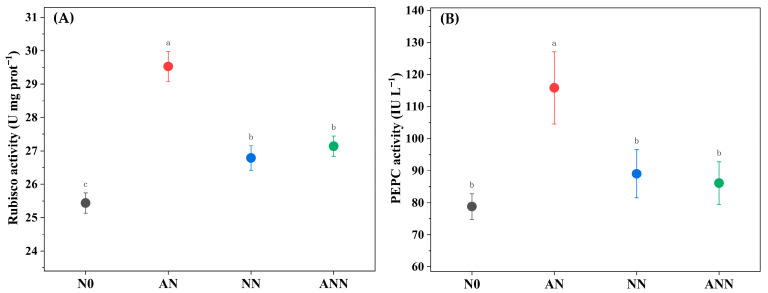
Effect of nitrogen forms treatments on Rubisco activity (**A**) and PEPC activity (**B**) in *H. altissima*. Different lower-case letters indicate significant differences between the measuring dates under the unfertilized (N0) treatment and the fertilized (AN, NN, ANN) treatment, respectively (*p* < 0.05) (n = 6).

The effects of N0, AN, NN, and ANN on Rubisco activity and PEPC activity were significant (*p* < 0.05) (Figure 5). The Rubisco activity and PEPC activity of the AN treatment was significantly higher than other treatments, and the Rubisco activity of the NN and ANN were significantly higher than N0 (*p* < 0.05) (Figure 5A). However, the PEPC activity in the N0, NN, and ANN treatments showed no significant differences (Figure 5B).

### 3.7. Within-Leaf Nitrogen Allocation Estimate

**Figure 6:**
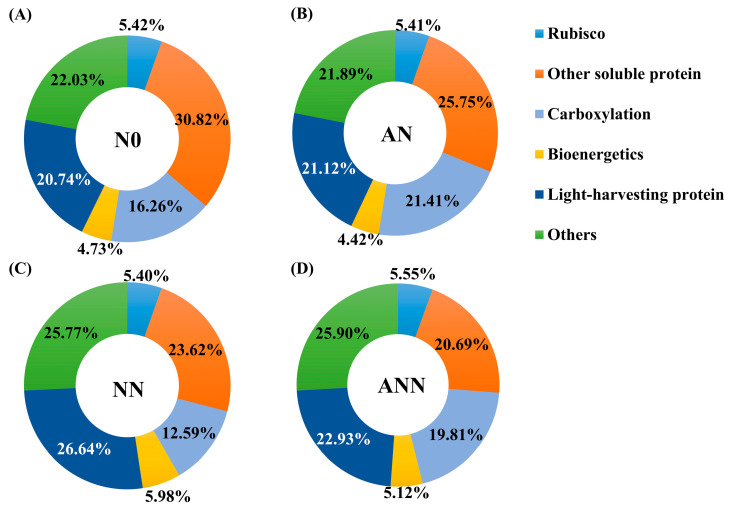
Effect of nitrogen forms treatments on the Nitrogen allocation in leaves of *H. altissima*. The data of percentages are the content of nitrogen in the corresponding components accounting for total leaf nitrogen content in *H. altissima*. (**A**–**D**) nitrogen partitioning in the unfertilized (N0) treatment and the fertilized (AN, NN, ANN) treatments. The size of pie chart indicates nitrogen content (*p* < 0.05) (n = 6).

The effects of different available nitrogen forms on the allocation of leaf nitrogen to different nitrogen components are shown in Figure 6. The rubisco, other soluble protein and carboxylation values expressed per unit leaf area were significantly higher under the AN treatment than under the N0, NN or ANN treatments (*p* < 0.05) (Table 1; Figure 6). Relative to the NN and ANN treatments, the AN treatment significantly increased the percentages of nitrogen allocated to rubisco (1.01% and −0.14%, respectively), other soluble protein (2.13% and 5.06%) and carboxylation (8.82% and 1.6%) proteins. Unexpectedly, no significant difference was found in N_B_ (Bioenergetics) between the NN and ANN treatments, but N_B_ was significantly higher in these treatments than in the N0 treatment (*p* < 0.05) (Table 1, Figure 6B). Relative to the AN and ANN treatments, the NN treatment significantly increased the percentages of nitrogen allocated to N_B_ (1.56% and 0.86%, respectively). Compared to the N0, AN and ANN treatments, N_L_ (Light-harvesting protein) increased under the NN treatment, while other nitrogen increased in ANN treatment (*p* < 0.05) (Table 2; Figure 6C,D).

**Figure 7:**
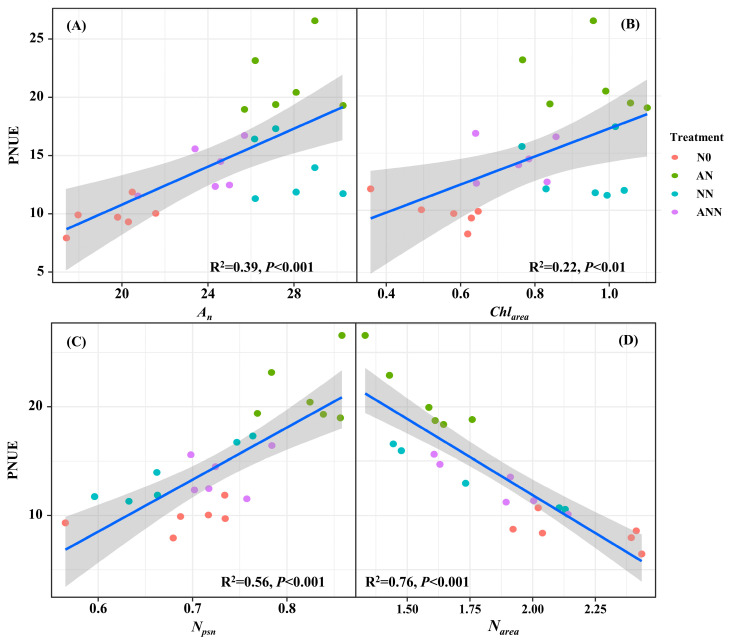
Relationships of photosynthetic N use efficiency (PNUE) with net CO_2_ assimilation rate (*A_n_*) (**A**), area-based chlorophyll content (*Chl_area_*) (**B**), photosynthetic N (*N_psn_*) (**C**) and area-based nitrogen content (*N_area_*) (**D**) in *H. altissima*. Relationships between variables were assessed using linear regression analysis.

Linear correlation analysis provided correlations of PNUE with photosynthetic responses of *A_n_* and *Chl_area_*, and nitrogen allocation of *N_psn_* and *N_area_* (Figure 7). Under different nitrogen forms treatments, PNUE are strongly shaped by *A_n_*, *Chl_area_* and *N_psn_* (*p* < 0.05). Meanwhile, there is a negative correlation between PNUE and *N_area_*, which will directly or indirectly affect the changes in PNUE.

### 3.8. PSII Quantum Efficiencies

**Figure 8:**
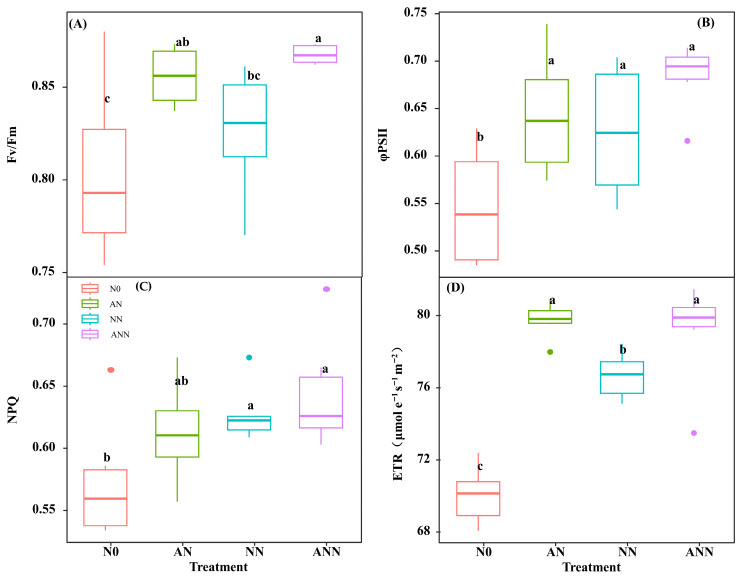
Effect of nitrogen forms treatments on the maximum quantum yield of PSII (Fv/Fm) (**A**), the effective quantum yield of PSII (φPSII) (**B**), non-photochemical quenching coefficient (NPQ) (**C**), and electron transport rate (ETR, μmol e^−1^ s^−1^ m^−2^) (**D**) in *H. altissima*. Different lower-case letters indicate significant differences between the measuring dates under the unfertilized (N0) treatment and the fertilized (AN, NN, ANN) treatment, respectively (*p* < 0.05) (n = 6).

*H. altissima* plants demonstrated a clear advantage in allocating nitrogen to photosynthetic components in their leaves across various nitrogen treatments. To explore the potential effects of nitrate and ammonium on PSII quantum efficiencies, we examined the relationship between these nitrogen forms and the plants’ photosynthetic performance. A strong, statistically significant positive linear correlation was observed between PNUE and *Chl_area_* in *H. altissima* (Figure 7B). Additionally, measurements of Fv/Fm, φPSII, non-photochemical quenching (NPQ), and electron transfer rate (ETR) were significantly higher under the AN and ANN treatments compared to the N0 and NN treatments (*p* < 0.05) (Figure 8).

## 4. Discussion

### 4.1. Effects of Different Forms of Nitrogen on the Nitrogen Source and Nitrogen Metabolic Enzyme Activity in H. altissima

Nitrogen metabolism is crucial for the synthesis of key proteins required for photosynthesis. The different forms of nitrogen sources in the soil can significantly influence the activity of enzymes related to plant nitrogen assimilation. Nitrate reductase (NiR) and nitrate reductase (NR) are involved in the reduction of NO_3_^−^ to NH_4_^+^, and they regulate this process through coupling mechanisms [29]. In this study, the inorganic nitrogen content in the soil significantly increased under AN treatment (Figure 1), which was directly related to changes in nitrogen metabolic enzyme activities. NN treatment significantly stimulated NR and NiR activities, consistent with previous research that indicates NR activity is mainly influenced by NO_3_^−^ concentration, promoting nitrification [15]. As NO_3_^−^ is converted into other forms of nitrogen, its availability decreases, but nitrogen in the soil continues to transfer to the leaves [30]. Our results indicate that AN and NN treatments significantly enhanced the activity of key nitrogen metabolic enzymes, such as glutamine synthetase (GS) and glutamate synthetase (GAGOT), with the AN treatment showing the most significant effect (Figure 2). This suggests that NH_4_^+^ play an important role in promoting nitrogen assimilation and improving overall plant metabolism, likely due to NH_4_^+^ preferentially entering the mesophyll cells to directly participate in nitrogen absorption and assimilation [31]. In particular, the increased GS activity under ammonium treatment may promote the synthesis of nitrogen in organic forms, thereby supporting the plant’s nitrogen economy. In higher plants, GSI and GOGAT assimilate NH_4_^+^ into amino acids for plant uptake [5]. The concentration of NH_4_^+^ is closely related to GSI and GOGAT enzyme activity [32], but under different nitrogen supply treatments, GSI enzyme activity was significantly higher under AN treatment compared to other nitrogen forms, which is consistent with previous research on rice plants [33]. The results of this study reveal the relationship between NO_3_^−^ and NH_4_^+^ supply and assimilation enzyme activity. According to our results, the activity of nitrogen isoenzymes was significantly increased after AN treatment.

### 4.2. Effects of Different Forms of Nitrogen on Leaf Morphological Traits and Gas Exchange in H. altissima

As is widely known, nitrogen is a vital nutrient for plant growth and development, and the form of nitrogen source directly affects leaf growth [34]. Leaf trait adjustments are often more important than biochemical characteristics in determining how a plant’s leaves adapt to the environment for photosynthesis [8,24]. Nitrogen promotes leaf area growth and maintains the leaf’s ability to absorb and utilize light energy, which helps increase net photosynthesis (*A_n_*) and photosynthetic nitrogen use efficiency (PNUE) [8,15]. Different nitrogen sources had significant effects on the photosynthetic performance of *H. altissima*. Under AN and NN treatments, leaf *A_n_* and stomatal conductance (*G_s_*) significantly increased (Figure 3), indicating better nitrogen allocation to the photosynthetic apparatus. In addition to the biochemical and physiological processes of photosynthesis, *G_s_* is also a key factor affecting CO_2_ assimilation. Our results indicate that the *G_s_* value of plants under AN treatment was higher than under other nitrogen source treatments (Figure 3B), which may be due to the role of ammonium and nitrate in regulating the opening and closing of stomata, as NH_4_^+^ is converted to nitrate through the anion transport system of nitrification, participating in the metabolism of guard cells [35]. The increase in *G_s_* value facilitated more CO_2_ absorption, and combined with better nitrogen allocation to photosynthetic proteins, this ultimately led to an increase in photosynthetic efficiency and PNUE. The accumulation of photosynthetic pigments in the leaves visually represented the photosynthetic efficiency, with *Chl_m_* significantly higher under AN treatment compared to other treatments, indicating that ammonium nitrogen significantly improved the photosynthetic efficiency of *H. altissima*. This enhancement in photosynthetic efficiency may be closely related to the effective distribution of nitrogen in the leaves, which is crucial for optimizing the photosynthetic apparatus of C_4_ plants. Key indicators of photosynthetic efficiency, such as Fv/Fm, φPSII, and ETR values [11], were significantly higher under NH_4_^+^ treatment, indicating an improvement in electron transport and light-harvesting efficiency (Figure 8). The high photosynthetic nitrogen use efficiency observed under nitrate conditions may be attributed to the optimized allocation of nitrogen in the photosynthetic apparatus, particularly the carboxylation system and the electron transport chain [36].

### 4.3. Effects of Different Forms of Nitrogen on Leaf Photosynthetic Performance and Photosynthetic Nitrogen Use Efficiency in H. altissima

The activity of key photosynthetic enzymes, such as Rubisco and PEP carboxylase (PEPC), is also affected by nitrogen sources. Under AN treatment, the activity of these enzymes, especially Rubisco and PEPC, was significantly increased, as they are core enzymes in carbon fixation in C_4_ plants [37]. The increase in enzyme activity may be due to the effective distribution of nitrogen to the photosynthetic apparatus, where nitrogen plays a crucial role in the synthesis and function of photosynthetic proteins [38]. Under ammonium nitrogen conditions, the increase in enzyme activity in *H. altissima* suggests that nitrogen from ammonium salts is more effectively allocated to photosynthetic proteins than nitrogen from nitrates, thereby improving the photosynthetic rate. *V_cmax_* as an indicator of Rubisco enzyme activity in the carbon fixation process of photosynthesis [35,36], was significantly increased by inorganic nitrogen sources. This indicates that ammonium nitrogen supply is closely related to the normal growth of *H. altissima* leaves. In this study, compared to N0, NN, and ANN treatments, the AN treatment showed higher *N_area_*, *A_n_*, and *Chl_area_* in *H. altissima*, with PNUE increasing by 26.12%, 11.02%, and 10.82%, respectively. In addition, PNUE in *H. altissima* showed a significant positive correlation with *A_n_*, *N_psn_*, and *Chl_area_*. Our results show that under AN treatment, nitrogen was preferentially allocated to the photosynthetic apparatus, especially to the chloroplasts, which is crucial for light absorption and energy transfer needed for photosynthesis [9,29]. This allocation promoted higher *Chl_area_* and improved photosynthetic efficiency. Furthermore, the higher PNUE observed under AN treatment showed a strong negative correlation with Narea (Figure 7D), further emphasizing the role of nitrogen allocation in photosynthetic efficiency. The induction of higher photosynthetic efficiency under AN treatment may be due to the higher efficiency of nitrogen distribution in the leaves, which enhances the performance of photosynthesis. This result supports that NH_4_^+^ are more effective in nitrogen distribution within the C_4_ photosynthetic apparatus, aiding in the photosynthetic optimization of *H. altissima*.

### 4.4. Effects of Different Forms of Nitrogen on Leaf Nitrogen Allocation and Trade-Offs in H. altissima

**Figure 9:**
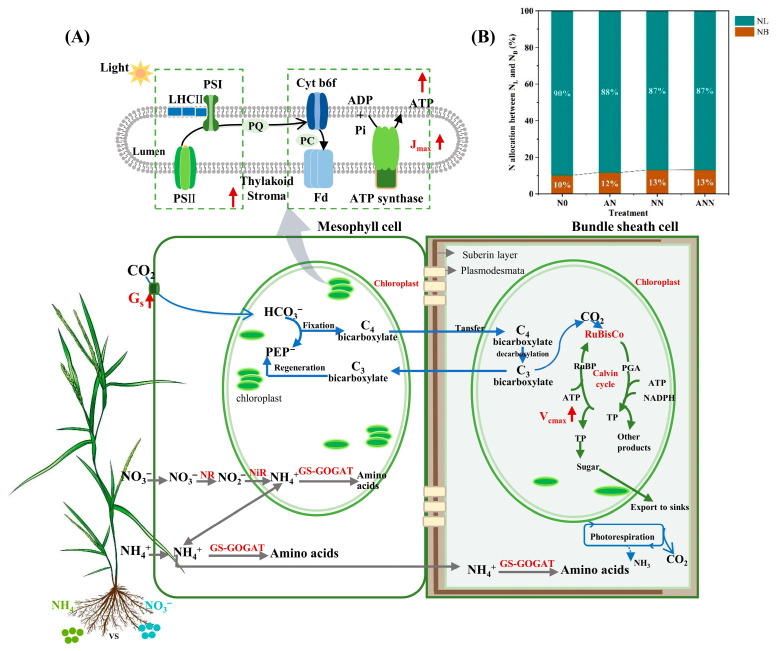
Effect of nitrogen form treatments on the change in nitrogen contents in the photosynthetic apparatus of leaves in *H. altissima*. (**A**) The percentage together indicates the increase (red arrows) on nitrogen in different photosynthetic apparatuses under AN compared to the N0, NN and ANN treatments. (**B**) The allocation of N between *PN_B_* and *PN_L_* within the thylakoid lumen under N0, AN, NN and ANN treatments.

The allocation of nitrogen within the leaves is critical for optimizing photosynthetic performance because it determines how nitrogen is distributed to the photosynthetic apparatus and other cellular structures. As with many plant species, the allocation of nitrogen within the leaves reflects the trade-off between growth and defense. Leaf nitrogen allocation reflects the trade-off within the leaf economics spectrum, where fast-growing species tend to allocate more nitrogen to growth metabolism at the expense of structural components [32]. This shift in nitrogen allocation may reflect the plant’s prioritization of photosynthesis while reducing nitrogen investment in structural functions. AN treatment led to a significant increase in the absolute content of soluble proteins and the proportion of Rubisco nitrogen (31.16%) (Table 2; Figure 6), a result consistent with previous research [4,6,10], who reported that 25–45% of leaf nitrogen is allocated to soluble proteins. Rubisco, a key enzyme in plant photosynthesis, accounts for 50% of soluble protein and 25% of leaf nitrogen [37]. In *H. altissima*, we found that AN treatment led to increased nitrogen allocation to Rubisco, reflected by lower bioenergetics and light-harvesting proteins, and higher carboxylation (Table 2; Figure 6). Compared to the NN and ANN treatments, N_B_/N_B+L_ decreased under the ANN treatment, while N_L_/N_B+L_ increased (*p* < 0.05) (Figure 9B). We found significant differences in nitrogen allocation to soluble proteins between AN treatment and other treatments, which is consistent with previous studies indicating that invasive species allocate more nitrogen to photosynthesis than native species, promoting growth and carboxylation [17,39]. The results of these studies suggest that more nitrogen is allocated to soluble proteins, sacrificing structural proteins [4,39]. These results suggest that under ammonium nitrogen conditions, nitrogen allocation helps improve nitrogen absorption and utilization, maximizing support for mesophyll cell photosynthesis. The changes in nitrogen investment strategies indicate that these components are crucial for ensuring the plant adapts to normal growth and physiological activities under inorganic nitrogen conditions.

Thus, we hypothesize that under AN treatment, *H. altissima* is usually in the “high return” zone of the leaf economics spectrum, with higher *A_n_*, *Chl_area_* and PNUE compared to other treatments, thereby allocating more nitrogen to leaf nitrogen pools related to photosynthesis and growth. Based on this analysis, species with larger investments in photosynthetic protein nitrogen typically show higher PNUE in many natural ecosystems [12,39,40]. In this study, *H. altissima* allocated 52.36% of its leaf nitrogen to the photosynthetic apparatus, consistent with previous studies on maize [36] and invasive plants [39]. According to our preliminary hypothesis, to determine if a plant is in the “high return” zone, the changes in leaf nitrogen allocation processes need to be assessed. In ecological models, nitrogen investment in the photosynthetic apparatus remains an important determinant of PNUE [15,41]. Photosynthesis is closely related to leaf nitrogen content, and nitrogen content is usually reflected by Calvin cycle proteins. Fast-growing plants allocate about two-thirds of their leaf nitrogen to the photosynthetic apparatus [6,12,14]. We found that the amount of nitrogen allocated to the photosynthetic apparatus was significantly positively correlated with PNUE (R^2^ = 0.56, *p* < 0.001). The different forms of nitrogen sources play a crucial role in optimizing the photosynthetic efficiency and nitrogen utilization efficiency of *H. altissima* (Figure 9). AN treatment promoted the effective allocation of nitrogen to the photosynthetic apparatus, enhancing enzyme activity, photosynthetic efficiency, and PNUE. Under AN treatment, higher stomatal conductance and optimized leaf nitrogen allocation further facilitated CO_2_ absorption and improved photosynthetic efficiency. The results highlight the importance of understanding nitrogen allocation dynamics for optimizing photosynthesis and plant growth, and suggest that increasing ammonium salt fertilizers in nitrogen-limited environments may be an effective strategy to optimize C_4_ plant photosynthesis and productivity.

## 5. Conclusions

Our study reveals that inorganic nitrogen sources play a pivotal role in shaping the PNUE, nitrogen assimilation, and nitrogen allocation in *H. altissima* leaves. The plants exhibited a clear adaptive response, optimizing nitrogen distribution within the leaves to enhance photosynthesis. This resulted in increased PNUE and biomass production during the growing season, especially in environments with high ammonium nitrogen levels. Under AN and ANN treatments, *H. altissima* allocated more nitrogen to rubisco and the carboxylation apparatus, thereby improving ETR. In addition, *Chl_m_* and NPQ increased, which helped to enhance the light protection capability. The treatment also shifted nitrogen allocation, with more nitrogen directed toward soluble proteins and the photosynthetic machinery. This reallocation suggests a trade-off between growth and absorption and utilization of nitrogen in *H. altissima*. Overall, our findings offer new insights into how inorganic nitrogen sources influence nitrogen dynamics in *H. altissima*, providing a deeper understanding of their nitrogen utilization strategies in the context of increasing nitrogen deposition. Understanding how *H. altissima* responds to inorganic nitrogen sources can guide the development of more targeted fertilization strategies. By knowing that ammonium nitrogen (AN) and ammonium nitrate nitrogen (ANN) enhance photosynthesis and biomass production, agriculturalists can optimize fertilizer types and application rates, especially in regions with high nitrogen deposition. This could improve crop yields without over-fertilizing, thus promoting both economic and environmental sustainability.

## Figures and Tables

**Table 1:** Effect of nitrogen forms treatments on the content of chlorophyll in *H. altissima*.

N Form Treatments	*Chl_a_* (mg g^−1^)	*Chl_b_* (mg g^−1^)	*Chl_m_* (mg g^−1^)	*Chl_area_* (g m^−2^)
N0	0.80 ± 0.06 a	0.52 ± 0.01 c	1.32 ± 0.06 c	0.55 ± 0.04 b
AN	0.73 ± 0.07 ab	0.97 ± 0.06 a	1.71 ± 0.05 a	0.95± 0.05 a
NN	0.79 ± 0.05 ab	0.69± 0.05 bc	1.48 ± 0.05 b	0.88± 0.07 a
ANN	0.59 ± 0.09 b	0.88 ± 0.11 ab	1.42 ± 0.05 bc	0.81 ± 0.04 a

Different lower-case letters indicate significant differences between the measuring dates under the un-fertilized (N0) treatment and the fertilized (AN, NN, ANN) treatment, respectively (*p* < 0.05) (n = 6).

**Table 2:** Effect of nitrogen forms treatments on the content of N compounds in *H. altissima*.

Parameters (mg m^−2^)	Nitrogen Forms Treatments			
	N0	AN	NN	ANN
Rubisco	83.38 ± 0.38 c	105.53 ± 0.36 a	97.63 ± 0.34 b	107.72 ± 0.29 a
Other soluble protein	473.66 ± 1.86 b	502.66 ± 2.45 a	427.35 ± 4.26 c	401.33 ± 4.28 d
Carboxylation	249.86 ± 6.41 c	418.06 ± 20.32 a	227.81 ± 18.91 d	384.15 ± 23.19 b
Bioenergetics	72.75 ± 1.82 c	86.37 ± 1.29 b	108.23 ± 2.86 a	99.22 ± 4.16 ab
Light-harvesting protein	318.74 ± 5.18 d	412.23 ± 7.82 c	482.08 ± 12.94 a	444.80 ± 11.37 b
Other nitrogen	338.6 ± 9.16 d	427.35 ± 21.38 c	466.18 ± 22.35 b	502.34 ± 29.34 a

Different lower-case letters indicate significant differences between the measuring dates under the un-fertilized (N0) treatment and the fertilized (AN, NN, ANN) treatment, respectively (*p* < 0.05) (n = 6).

## Data Availability

The datasets generated for this study are available on request to the corresponding author.

## Abbreviations

*A_n_*	Assimilation rate
*G_s_*	Stomatal conductance
*C_i_*	Internal CO_2_ concentration
*WUE*	Water-use efficiency
*V_cmax_*	Maximum rate of Rubisco carboxylation
*J_max_*	Maximum rate of electron transport
*LMA*	A leaf mass per unit leaf area
*N_area_*	A leaf nitrogen content per unit leaf area
*N_C_*	Nitrogen contents in carboxylation
*N_B_*	Nitrogen contents in bioenergetics
*N_L_*	Nitrogen contents in light harvesting components
N_psn_	Nitrogen contents in photosynthetic apparatus
PNUE	Photosynthetic nitrogen use efficiency
Fv/Fm	The maximum quantum yield of PSII
NPQ	The non-photochemical quenching coefficient
φPSII	The effective quantum yield of PSII
ETR	The electron transport rate
